# Effect of TiO_2_ Coating on Structure and Electrochemical Performance of LiNi_0.6_Co_0.2_Mn_0.2_O_2_ Cathode Material for Lithium-Ion Batteries

**DOI:** 10.3390/ma17246222

**Published:** 2024-12-19

**Authors:** Lin Li, Zhongyu Li, Zhifan Kuang, Hao Zheng, Minjian Yang, Jianwen Liu, Shiquan Wang, Hongying Liu

**Affiliations:** 1 School of Chemical Engineering, Guizhou University of Engineering Science, Bijie 551700, China; honglinymj@163.com; 2 Collaborative Innovation Center for Advanced Organic Chemical Materials Co-Constructed by the Province and Ministry, Ministry of Educational Key Laboratory for the Synthesis and Application of Organic Functional Molecules, College of Chemistry and Chemical Engineering, Hubei University, Wuhan 430062, China; 202321106011493@stu.hubu.edu.cn (Z.L.); kzf18720677606@163.com (Z.K.); zhengaho1986@126.com (H.Z.); jianwen@hubu.edu.cn (J.L.); liuhy@hubu.edu.cn (H.L.); 3 Hubei Three Gorges Laboratory, Yichang 443008, China

**Keywords:** LiNi_0.6_Co_0.2_Mn_0.2_O_2_, TiO_2_, surface coating, cyclic performance, lithium-ion batteries

## Abstract

High-nickel ternary LiNi_0.6_Co_0.2_Mn_0.2_O_2_ (NCM622) is a promising cathode material for lithium-ion batteries due to its high discharge-specific capacity and energy density. However, problems of NCM622 materials, such as unstable surface structure, lithium–nickel co-segregation, and intergranular cracking, led to a decrease in the cycling performance of the material and an inability to fully utilize high specific capacity. Surface coating was the primary approach to address these problems. The effect of TiO_2_ coating prepared by the sol–gel method on the performance of LiNi_0.6_Co_0.2_Mn_0.2_O_2_ was studied, mainly including the morphology, cell structure, and electrochemical properties. LiNi_0.6_Co_0.2_Mn_0.2_O_2_ was coated by TiO_2_ with a thickness of about 5 nm. Compared with the pristine NCM622 electrode, the electrochemical performance of the TiO_2_-coated NCM622 electrodes is improved. Among all TiO_2_-coated NCM622, the NCM622 cathode with TiO_2_ coating content of 0.5% demonstrates the highest capacity retention of 89.3% and a discharge capacity of 163.9 mAh g^−1^, in contrast to 80.9% and145 mAh g^−1^ for the pristine NCM622 electrode, after 100 cycles at 0.3 C between 3 and 4.3 V. The cycle life of the 5 wt% TiO_2_-coated NCM622 electrode is significantly improved at a high cutoff voltage of 4.6 V. The significantly enhanced cycling performance of TiO_2_-coated NCM622 materials could be attributed to the TiO_2_ coating layer that could block the contact between the material surface and the electrolyte, reducing the interface side reaction and inhibiting the transition metal dissolution. At the same time, the coating layer maintained the stability of layered structures, thus reducing the polarization phenomenon of the electrode and alleviating the irreversible capacity loss in the cycle process.

## 1. Introduction

With the increasing consumption of fossil fuels, the development of a green industrial revolution and the use of clean, renewable energy to achieve carbon substitution have become an inevitable trend, while the development and promotion of renewable energy are also inseparable from reliable energy storage devices [1]. As a kind of high efficiency and cheap energy storage equipment, lithium-ion batteries (LIBs) are widely used in various industries, especially electric vehicles and portable electronics, with the advantages of high energy density, small size, light weight, no memory effect, low self-discharge, and long life compared with traditional secondary batteries [2,3]. The cathode materials play a crucial role in the electrochemical performance of LIBs, so good stability, high operating voltage, safety, low cost, and high capacity cathode material have to be developed, matching high-performance LIBs [4,5].

High nickel ternary LiNi_0.6_Co_0.2_Mn_0.2_O_2_ (NCM622) is a promising cathode material for lithium-ion batteries due to its high discharge specific capacity and energy density [6]. However, there are still some key challenges in the production, cycling, and multiplication performance of this material (such as low coulombic efficiency, low thermal stability, low structural stability, microcracks, interfacial side reactions, etc.), which limits their further application [7]. This is mainly attributed to (1) Li^+^/Ni^2+^ cation mixing problems due to similar ionic radii. This can trigger a phase transition and reduce the capacity of LiNi_0.6_Co_0.2_Mn_0.2_O_2_ [8]. (2) Li^+^ on the surface of LiNi_0.6_Co_0.2_Mn_0.2_O_2_ reacts with air to form alkaline impurities such as Li_2_CO_3_ and LiOH, which accelerates the decomposition of the electrolyte and the formation of HF, causing damage to the electrode [9]. In addition, due to the presence of high-valent Ni ion, the surface structure of the cathode material is unstable and is prone to side reactions with the electrolyte, while the grain boundaries of the cathode particles are prone to cracking, which makes the capacity of lithium-ion batteries decay more quickly [10,11].

A large number of studies have shown that suitable surface modification can play a role in stabilizing the surface chemistry and structure of the material and is also an effective method to enhance the electrochemical performance of ternary materials [12]. Coating materials have proven to be significantly effective, including non-metallic C [13], metal oxides (TiO_2_, Al_2_O_3_) [14], MgHPO_4_ [15], ZrO_2_ [16,17], Li_2_SnO_3_ [18], fluorides AlF_3_ [19], LaF_3_ [20], AlPO_4_ [21], Li_3_PO_4_ [22], Mn_3_(PO_4_)_2_ [23], etc. A portion of the work on NCM-622 material coating modification is shown in Table S1. Yang et al. [24] found that the side reaction between cathode material and electrolyte was effectively suppressed due to the appropriate amount of Sm_2_O_3_ coating, and the 0.5 wt% Sm_2_O_3_ NCM material showed higher cycling stability (88.97% at 1 C after 200 cycles). Under the influence of the synergistic effect of ion doping and LiNbO_3_ coating, the LiNbO_3_ coated Mg-doped NCM-622 cathode material exhibits excellent electrochemical performance. Thanks to the LiNbO_3_ coating, which reduces the effect of surface side reactions, and the Mg^2+^ doping, which stabilizes the structure of the material, the cathode material exhibits excellent long-cycle performance and large-multiplication performance under high-voltage operating conditions [25].

TiO_2_ is a competitive cathode coating material due to its unique properties, including abundance, structural stability, and electrochemical inertness [26]. Li et al. [27] showed that an appropriate amount of TiO_2_ coating can effectively inhibit the interfacial reaction between the electrode material and electrolyte to improve the structural stability of the material. Wang et al. [28] reported that the TiO_2_ coating modification treatment could effectively alleviate the internal cracks, phase transition, and main structure change in single crystal LiNi_0.6_Co_0.2_Mn_0.2_O_2_ (SC-NCM) material, and the capacity retention rate of SC-NCM electrode material was 87.4% after 200 cycles. Qin et al. [29] reported the preparation of amorphous TiO_2_-coated LiNi_0.6_Co_0.2_Mn_0.2_O_2_ cathode materials using the atomic layer deposition (ALD) technique. The amorphous TiO_2_ coating can inhibit the dissolution of metal ions in the LiNi_0.6_Co_0.2_Mn_0.2_O_2_ material and favor the lithium diffusion of the oxide (187.7 mA hg^−1^ at 0.1 C for the initial discharge capacity). Compared with the pure substance, the modified LiNi_0.65_Co_0.15_Mn_0.2_O_2_ material showed excellent cycling performance and high-temperature performance through nano-TiO_2_ modified treatment. This is mainly due to the fact that the Nano-TiO_2_ cladding treatment mitigates the side reactions at the electrode/electrolyte interface, suppresses the irreversible H2/H3 phase transition, and slows down the structural pulverization of the material [30]. Mo et al. [31] obtained TiO_2_-modified LiNi_0.6_Co_0.2_Mn_0.2_O_2_ materials by wet capping. It was found that the Ti ions in the TiO_2_ capping layer formed a diffusion layer with different Ti^4+^ concentrations. This diffusion layer with different Ti^4+^ concentrations accelerated the electron and Li^+^ migration rates and enhanced the interfacial stability and mechanical strength of the material.

In this study, a uniform TiO_2_ coating layer was slowly generated on the surface of coated LiNi_0.6_Co_0.2_Mn_0.2_O_2_ by slowly hydrolyzing in air, tetrabutyl titanate as a raw material for titanium. The effects of the concentration of tetrabutyl titanate on the morphology, structure, and electrochemical properties of the products were investigated. It was found that tetrabutyl titanate could form a thin TiO_2_ layer on the surface of high nickel material. Compared with the unmodified sample, the cycling stability of the TiO_2_-modified LiNi_0.6_Co_0.2_Mn_0.2_O_2_ sample was significantly improved. The specific capacity of 5 wt% TiO_2_-coated NCM electrode only decreased from 183.5 mAh g^−1^ to 163.9 mAh g^−1^, and the capacity retention rate was increased to 89.3%. The TiO_2_ coating effectively reduces the occurrence of side reactions at the electrode/electrolyte interface, lowers the interfacial impedance, and reduces the polarization of TiO_2_-coated NCM during the cycling process.

## 2. Materials and Methods

All samples used are analytically pure. LiNi_0.6_Co_0.2_Mn_0.2_O_2_ cathode materials were synthesized using high-temperature solid phase sintering. The Ni_0.6_Co_0.2_Mn_0.2_(OH)_2_ precursors (Hunan Zhongwei Technology Co., Ltd., Xiangtan, China) and LiOH·H_2_O were weighed according to the molar ratio of 1.03:1 and ground in an agate mortar for 0.5 h. The mixture was sintered at 480 °C for 6 h and then at 750 °C for 12 h with a temperature increase rate of 5 °C/min under an O_2_ atmosphere. The sintered product is the NCM622 cathode material.

**Figure 1:**
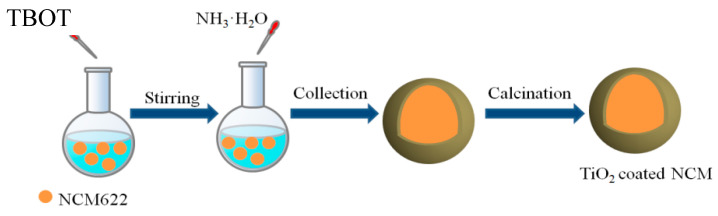
Schematic illustration of synthesis procedure for TiO_2_-NCM622.

Figure 1 shows the preparation of TiO_2_-coated NCM622. An amount of 2 g of NCM622 and tetrabutyl titanate (TBOT, 0.2557 g, 0.6393 g and 1.0655 g corresponds to 2.0, 5.0, and 8.0 wt% TiO_2_) were homogeneously dispersed in anhydrous ethanol, then 0.4 mL of NH_3_·H_2_O was added dropwise and stirred for 2.5 h. Then, the obtained powder was collected by centrifugation and washed with deionized water and ethyl alcohol three times, and the above mixture was dried under vacuum at 60 °C for 12 h. Finally, the mixture was annealed at 500 °C for 4 h to produce coating samples. The samples were recorded as 2 wt%, 5 wt%, and 8 wt% TiO_2_-NCM, respectively, according to the addition content of TiO_2_.

## 3. Results and Discussion

**Figure 2:**
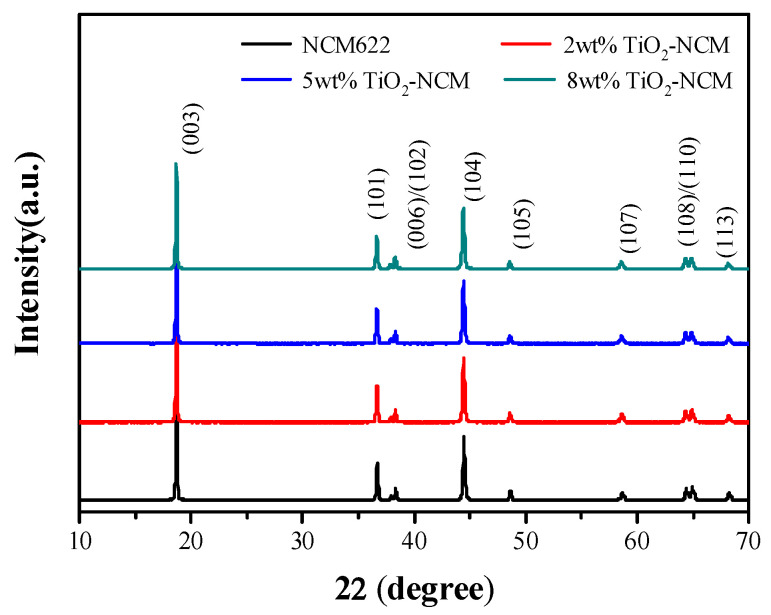
XRD patterns of NCM622 and TiO_2_ coated NCM samples.

Figure 2 shows the X-ray diffraction (XRD) patterns of NCM622 and three modified samples with different amounts of TiO_2_ coating. As shown in Figure 2, the diffraction peaks of all samples match well with the standard PDF card (JCPDS, No. 87-1560), which corresponds to the R3m space group of the hexagonal α-NaFeO_2_ structure [32]. The more obvious splitting of the (006)/(012) and (018)/(110) peaks observed at 38° and 65°, respectively, suggested that the material has a good lamellar structure and also implies that the original structure of the material is well preserved by the capping method used [19]. In addition, no diffraction peaks of TiO_2_ were observed in the XRD spectra of the modified samples, which may be due to the small amount of cladding used [20]. It is also observed that compared with the NCM622 bulk sample, the (003) peak and the (104) peak of the modified sample have a leftward shift, and there is no obvious change in the peak intensity of the diffraction peaks, which suggests that for the TiO_2_-coated sample, a small amount of Ti^4+^ may have diffused into the structure of the NCM622 bulk phase, broadening the (003) crystal plane spacing [Figure S1a,b] [33].

The cell structure parameters of the pure NCM622 and TiO_2_-coated NCM samples were obtained by using a JADE (MDI JADE 6.5) software analyzer. The results can reflect the degree of cation mixing and exclusion in nickel-rich materials. The cell parameters of the pure NCM622 and TiO_2_-coated NCM samples are shown in Table 1. The I_003_/I_104_ value and c/a value are used to assess the degree of Ni/Li mixing in nickel-rich materials. The I_(003)_/I_(104)_ values of bare MCM622, 2 wt%, 5 wt%, 8 wt% TiO_2_-NCM are 1.65, 1.81, 1.83, 1.88, respectively, as shown in Table 1. Compared with the NCM622 bulk sample, the c-axis length of the TiO_2_-coated NCM sample is significantly larger, and the Li/Ni mixing degree is increased, which further confirms that part of Ti^4+^ doping enters into the bulk structure of NCM622, and part of Ni^2+^ in the transition metal sites enters into Li^+^ due to the charge equilibrium, which increases the Li/Ni mixing degree of the material [14]. In addition, when the c/a value is greater than 4.9, the material is considered to have a complete laminar structure. As can be seen from Table 1, the c/a values of all the samples are greater than 4.9, so it can be determined that four kinds of materials have a complete laminar structure [25]. It is generally believed that a high Li/Ni miscibility will hinder the interlayer Li^+^ transport during the charge/discharge process; however, a moderate Li/Ni miscibility can help improve the electrochemical performance of the material [22]. 

The (003) peak of TiO_2_-coated NCM samples shifts toward a lower two-theta angle compared with pristine NCM622 bulk, as shown in Figure S1. These subtle structural variations after TiO_2_ coating are mainly due to the larger size of the Ti^4+^ in an octahedral environment (r_Ti4+_ = 0.60 Å) as compared to that of Ni^3+^ (r_Ni3+_ = 0.56 Å), Co^3+^ (r_Co3+_ = 0.54 Å), and Mn^4+^ (r_Mn4+_ = 0.53 Å), which suggests the partial Ti^4+^ incorporated into the NCM622 lattice [31,32]. 

**Figure 3:**
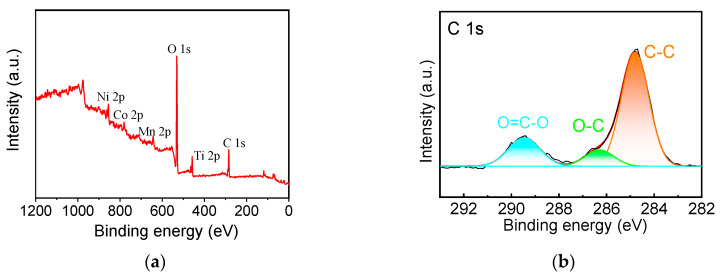

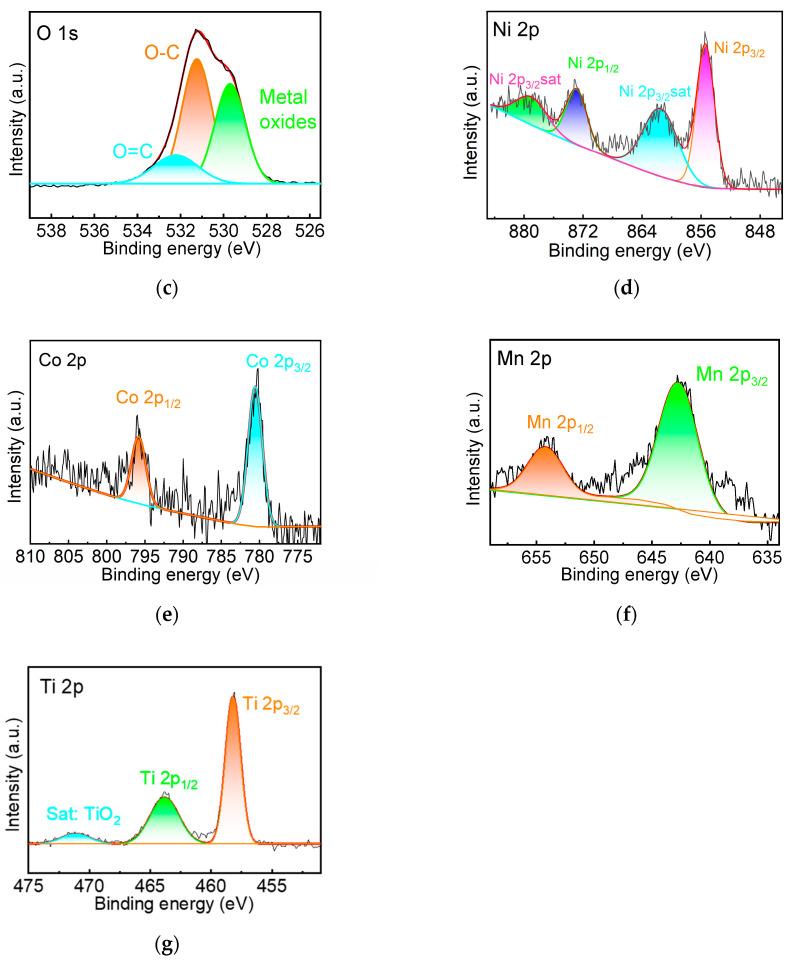
The XPS spectra of 5 wt% TiO_2_-NCM: (**a**) survey spectrum, (**b**) C 1s, (**c**) O 1s, (**d**) Ni 2p, (**e**) Co 2p, (**f**) Mn 2p, (**g**) Ti 2p.

Figure 3 shows the XPS full spectrum of the 5 wt% TiO_2_-coated NCM sample, from which it can be observed that there are characteristic peaks of Ni, Co, Mn, C, and O elements on the surface of the material, and there are also characteristic peaks of Ti element on the surface of the 5 wt% TiO_2_-coated NCM sample. Figure 3c shows the C 1s spectra, from which it can be seen that three peaks located at binding energies of 284.8, 286.4, and 289.3 eV were fitted to three peaks, which are attributed to sp2 bonded C-C, C-O, and C=O, respectively. The Ni 2p_1/2_ and Ni 2p_3/2_ peaks in the 5 wt% TiO_2_-coated NCM are located at 872.68 eV and 855.08 eV, respectively; the Mn 2p_3/2_ and Mn 2p_1/2_ peaks are located at 642.58 eV and 653.98 eV, respectively; and the characteristic peaks at the positions of 780.28 and 795.18 eV correspond to Co 2p_3/2_ and Co 2p_1/2_, respectively [30]. It can be seen that the TiO_2_-coated NCM622 modified material did not change the valence states of Ni, Mn, and Co elements. The characteristic peaks at positions 529.3 eV, 531.6 eV, and 532.5 eV corresponded to O1s. The characteristic peaks of Ti 2p_3/2_, Ti 2p_1/2_, and TiO_2_ appeared in the 5 wt% TiO_2_-coated NCM sample at positions located at 458.2 eV, 463.9 eV, and 472 eV (Figure 3g), which indicated the presence of Ti^4+^ on the surface of the 5 wt% TiO_2_, which was in agreement with the previous study [31]. Figure S2 is the XPS spectra of bare NCM622. Figure S2a is the survey spectrum of bare NCM622;there are characteristic peaks of Ni, Co, Mn, C, and O elements on the surface of the material. Figure S2b–d are characteristic peaks of Ni 2p, Co 2p, and Mn 2p, respectively, showing similar binding energies with the 5 wt% TiO_2_-coated NCM, except without the Ti element. 

**Figure 4:**
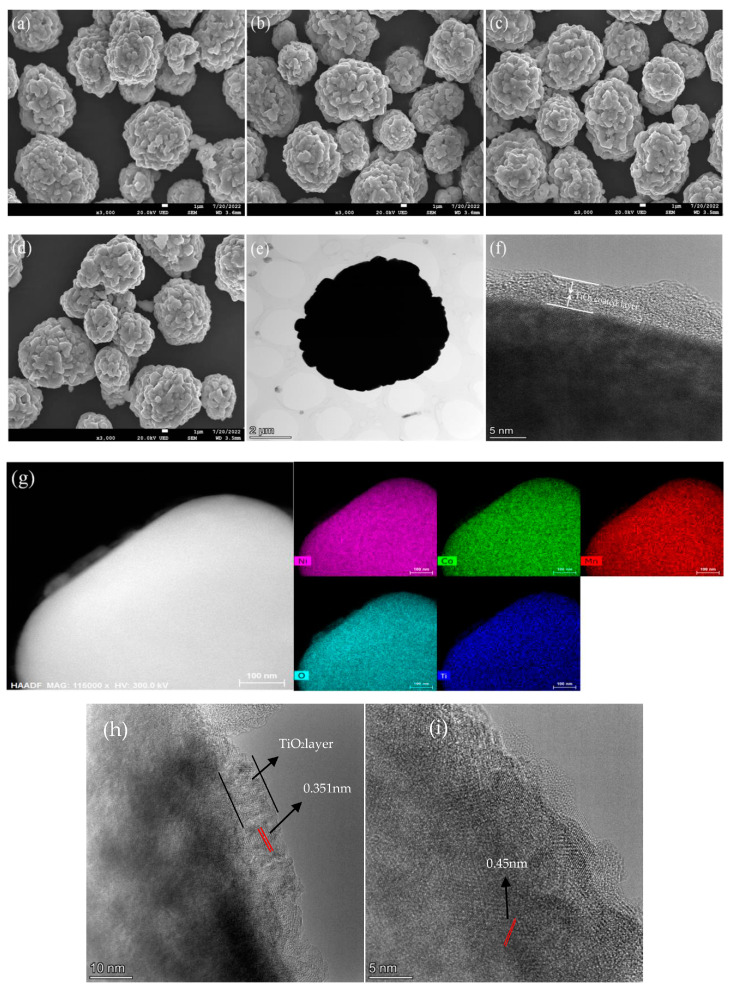
The SEM images of (**a**) NCM622, (**b**) 2 wt% TiO_2_-NCM, (**c**) 5 wt% TiO_2_-NCM, and (**d**) 8 wt% TiO_2_-NCM. (**e**) TEM image of 5 wt% TiO_2_-NCM; (**f**) HRTEM image; (**g**) FE-SEM elemental mapping of 5 wt% TiO_2_-NCM microspheres. (**h**,**i**) HRTEM images of 5 wt% TiO_2_-NCM.

Figure 4a–d shows the SEM images of NCM622 and TiO_2_-coated materials with different TiO_2_ amounts. From Figure 4, it can be seen that the particles take on the spherical morphology with a size of 5–10 µm. These microspheres are assembled by particles, as shown in Figure 4a–d. Figure 4e,f is the typical TEM images of a microsphere for 5 wt% TiO_2_-NCM. It indicates that TiO_2_ coating has no significant effect on the particle size and shape of NCM622. As shown in Figure 4e,f, the surface of the 5 wt% TiO_2_-NCM sample is relatively smooth and clean, the particle boundaries are obvious, and the surface of the 5 wt% TiO_2_-NCM sample has a relatively uniform titanium dioxide coating layer with a thickness of about 5 nm. The inert TiO_2_ coating layer can effectively reduce the contact between the material and the electrolyte, which can reduce the side reactions on the surface of the material and give the cathode material an excellent surface structure and stability [26]. The elemental mapping of 5 wt% TiO_2_-NCM microsphere indicates that the distribution of Ni, Co, Mn, O, and Ti elements are on the surface of the sample, as shown in Figure 4g. Additionally, the elemental mapping (Figure 4g) shows that the Ti and O are distributed homogeneously throughout the surface and enter the particle, while most of the Ni, Co, and Mn elements are distributed on the bulk and a few on the layer part of the particle [31]. Figure 4h,i shows the HRTEM images of 5 wt% TiO_2_-NCM, demonstrating the TiO_2_ coating layer with obvious lattice spacing and NCM interface lattice of 0.45 nm within the TiO_2_-NCM sample.The lattice spacing is about 0.351 nm, corresponding to the (101) plane of anatase phase TiO_2_. According to the HRTEM images of TiO_2_-coated material, the TiO_2_ coating layer, the pristine material, and the coating process indicate that there is a joint connection between the TiO_2_ coating layer and the pristine material.

**Figure 5:**
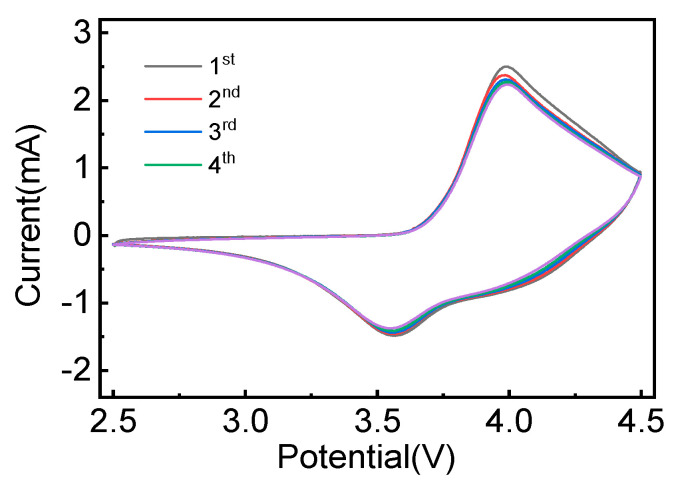
CV curves of 5 wt% TiO_2_-NCM.

Figure S3 and Figure 5 show the cyclic voltammetry (CV) curves of the NCM622 and 5 wt% TiO_2_-NCM samples. In the first cyclic voltammetry curve of the NCM622 and 5 wt% TiO_2_-NCM, the oxidation process of Ni^2+^/Ni^4+^ and the anodic delithiation reaction occurred at the oxidation peak position of 3.9~4.1 V, while the reduction process of Ni^2+^/Ni^4+^ and the anodic lithium embedding reaction occurred at the reduction peak position of 3.55~3.7 V. Compared with the NCM622 sample, the reproducibility of the CV curves of the 5 wt% TiO_2_-NCM material is better as the number of scanning turns increases, indicating that the 5 wt% TiO_2_-NCM material has excellent cycling stability. In addition, the oxidation peak moves from the high-voltage position in the first turn to the low-voltage direction in the second and third turns, indicating that the first charging and discharging process is an electrochemical activation process for the material to carry out in order to make the cation arrangement more orderly. This is advantageous for the detachment and embedding of Li ions in the subsequent electrochemical reaction process, which is manifested by the subsequent decrease in the oxidation peak voltage in the second and third turns [30,31,32].

**Figure 6:**
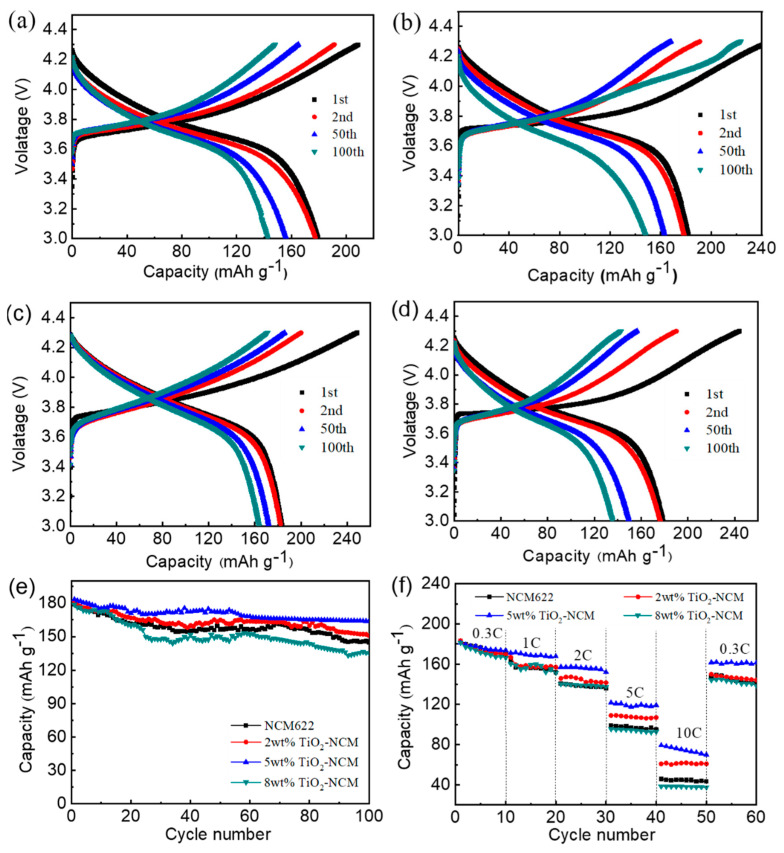
The charge-discharge curves of (**a**) NCM622, (**b**) 2 wt% TiO_2_-NCM, (**c**) 5 wt% TiO_2_-NCM, and (**d**) 8 wt% TiO_2_-NCM samples. (**e**) The long-term cycle of NCM, 2 wt% TiO_2_-NCM, 5 wt% TiO_2_-NCM, and 8 wt% TiO_2_-NCM samples at 0.3 C. (**f**) Rate performance of NCM, 2 wt% TiO_2_-NCM, 5 wt% TiO_2_-NCM, and 8 wt% TiO_2_-NCM samples at different current densities.

The constant current charge–discharge curves, cyclic stability, and rate performance of pristine NCM622, 2 wt%, 5 wt%, and 8 wt% TiO_2_-NCM samples are shown in Figure 6a–d. In Figure 6a–d, all samples exhibit a plateau region in the first discharge curve, related to the redox reaction between Ni^2+^/Ni^4+^ and Co^3+^/Co^4+^ brought about by deintercalation and intercalation of Li^+^ ions in the crystal lattice. The first cyclic discharge capacity of the 5 wt% TiO_2_-NCM at 0.3 C is 183.5 mAh g^−1^, which is higher than that of the pristine NCM622 of 179.2 mAh g^−1^, and the average Coulombic efficiency (CE) of 5 wt% TiO_2_-NCM can reach 100%. After 100 cycles, the pristine NCM622 shows a discharge capacity of 145 mAh g^−1^, whereas 5 wt% TiO_2_-NCM can maintain a discharge capacity of 163.9 mAh g^−1^. The above results indicate that the 5 wt% TiO_2_-NCM has an excellent reversible specific capacity compared to the pristine NCM622. Furthermore, in Figure 6a–d, the voltage plateau of NCM811 also has a more substantial decline with the increase in the number of cycle turns. The charge–discharge curves of 2 wt% and 5 wt% TiO_2_-NCM electrode materials are more compact, and the voltage and specific capacity decay more slowly. However, the voltage plateau of the 8 wt% TiO_2_-NCM electrode material also has a large decline with the increase in the coating amount, which may be due to the drastic side reactions in the material with the increase in the TiO_2_ coating amount [31]. This proves that the moderate amount of TiO_2_ coating has a better protective effect on the material, isolating the direct contact between the active substance and the electrolyte, reducing the occurrence of side reactions, lowering the interfacial impedance, and reducing the polarisation of TiO_2_-NCM electrode material [28].

Figure 6e shows the cycling stability of the pristine NCM622, 2 wt%, 5 wt%, and 8 wt% TiO_2_-NCM samples after 100 cycles at 0.3 C. The cycling stability after 100 cycles at 0.3 C is shown in Table S1, where the 5 wt% TiO_2_-NCM622 consistently maintains higher capacity and better cycling stability. For the NCM622 electrode, the reversible specific capacity decreases to 145 mAh g^−1^ after 100 cycles, whereas the 5 wt% TiO_2_-NCM electrode still releases a reversible specific capacity of 163.9 mAh g^−1^ after 100 cycles, with a total capacity retention rate of 89.3%. The initial coulombic efficiency (ICE) of NCM622, 2 wt% TiO_2_-NCM, 5 wt% TiO_2_-NCM, and 8 wt% TiO_2_-NCM samples are shown in Table S1, showing that the four kinds of materials exhibit ICE of 80.9, 83.0, 89.3, 75.7%, respectively. In general, the TiO_2_-coated NCM electrodes exhibit higher ICEs compared with bare NCM, except that the 8 wt% TiO_2_-NCM sample behaves with lower ICE, as shown in Table S1. This indicates that more TiO_2_ coating content is not beneficial to the ICE improvement. Figure S4 shows the CE of the four kinds of materials within 100 cycles. In general, the CEs of TiO_2_-coated NCM electrodes are close to 100% except for several cycles. The multiplicity performance of the samples is shown in Figure 6f. In general, the TiO_2_-NCM electrodes exhibit better rate performance compared with bare NCM622. The 5 wt% TiO_2_-NCM sample exhibits the best rate performance, with a discharge-specific capacity of an average of 70 mAh g^−1^ at a rate of 10.0 C, compared with ~35 mAh g^−1^ for bare NCM. The as-synthesized 5 wt% TiO_2_-NCM exhibits the best electrochemical performance compared with other TiO_2_-coated NCM electrodes, as shown in Table 2. The 8 wt% TiO_2_-NCM sample exhibits the worst rate performance, indicating that the larger coating content of TiO_2_ is diverse to the enhancement of electrochemical performance. The appropriate TiO_2_ coating of NCM622 has the lowest cation mixing and highly ordered lamellar structure, which is conducive to the dislodgement and embedding of Li-ions, and the diffusion kinetics of Li-ion is faster; thus, the sample has the best rate performance [26].

**Figure 7:**
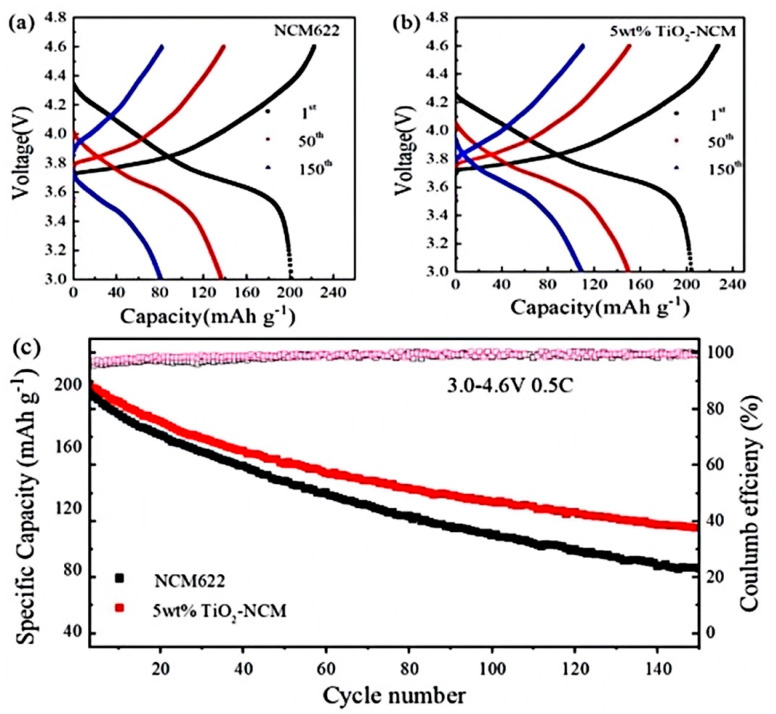
The charge-discharge curves of (**a**) NCM622, (**b**) 5 wt% TiO_2_-NCM sample, and (**c**) cyclic curve under 0.5 C and 4.6 V conditions.

Figure 7 shows the cycling performance of NCM622 and 5 wt% TiO_2_-NCM622 materials (at 3.0–4.6 V, 0.5 C). As can be seen from the figure, after 150 cycles, the 5 wt% TiO_2_-NCM622 cathode material still releases a discharge-specific capacity of 107.3 mAh g^−1^, which is higher than the unmodified NCM622 material (80.8 mAh g^−1^). At the beginning of the cycle, the two materials have similar cycle tendencies. As the number of cycles is increased, the capacity of unmodified NCM622 decays more seriously, and the voltage plateau gradually disappears during the cycling process, resulting in a more serious polarisation phenomenon. In contrast, 5 wt% TiO_2_-NCM622 electrode material shows a smaller degree of electrochemical performance degradation. This is mainly attributed to the fact that the appropriate amount of TiO_2_ coating layer avoids the contact between the active material and the electrolyte, inhibits the dissolution of transition metals and the side reaction with the electrolyte, stabilizes the material structure, reduces the dynamic mixing of cations, and improves the cycling and multiplicity performance of the material [34]. 

A comparison of performance with various NCM cathodes reported previously by other researchers is summarized in Table S2, highlighting the better electrochemical performance of TiO_2_-coated NCM electrodes in this study.

**Figure 8:**
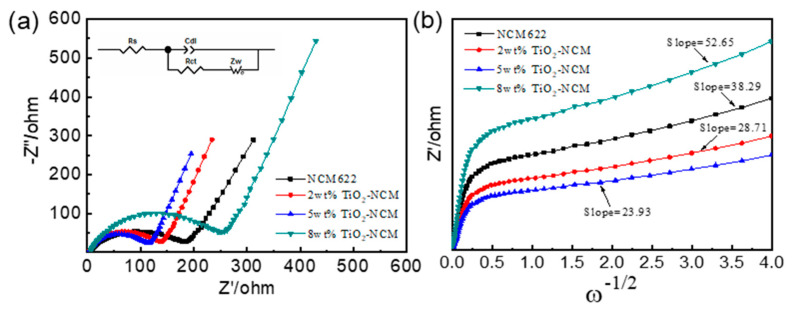
(**a**) Nyquist plots of NCM, 2 wt% TiO_2_-NCM, 5 wt% TiO_2_-NCM, and 8 wt% TiO_2_-NCM electrodes (the inset is an equivalent circuit.); (**b**) the linear fitting diagram of the Warburg impedance.

Electrochemical impedance spectroscopy (EIS) was performed to analyze the kinetics of NCM622 and TiO_2_-modified NCM materials. As shown in Figure 8a, the Nyquist plots of pristine NCM622, 2 wt%, 5 wt%, and 8 wt% TiO_2_-NCM samples all contain two cross-sections, which are the high-frequency region and low-frequency region cross-sections, respectively [35]. The semicircle in the high-frequency region indicates the charge transfer resistance (R_ct_); the diagonal line in the low-frequency region indicates the diffusion impedance of lithium ions inside the cathode material [36,37]. The specific R_ct_ values are shown in Table S2. It can be found that the 5 wt% TiO_2_-NCM electrode has the smallest charge transfer resistance. The straight line in the low-frequency region corresponds to the Warburg resistance (A_w_) of the diffusion of Li-ion inside the electrode, and the larger the slope is, the larger the Warburg resistance, as shown in Figure 8b. The Warburg coefficients of the four electrodes, NCM622, 2 wt% TiO_2_-NCM, 5 wt% TiO_2_-NCM, and 8 wt% TiO_2_-NCM, are 191, 142, 113, and 258 Ω s^−1/2^, respectively, by fitting, which proves that the 5 wt% TiO_2_-NCM has a faster electrochemical kinetics. The appropriate amount of TiO_2_ coating layer avoids the contact between the active material and the electrolyte, inhibits the dissolution of transition metals and the side reaction with the electrolyte, stabilizes the material structure, reduces the dynamic mixing of cations, and improves the electrochemical performance of the material [38,39]. 

The fade mechanism of electrochemical properties for bare NCM and improvement mechanism for TiO_2_-coated NCM materials need to be conducted through several characterization techniques, especially at a higher cutoff voltage of 4.6 V. Further work will also include the combination of some modification strategies for improving the electrochemical performance of NCM materials.

## 4. Conclusions

In summary, TiO_2_-coated NCM material cathode materials were prepared by a simple hydrolysis reaction. The effects of different amounts of TiO_2_ coating on the morphology, structure, and electrochemical properties of the products were studied.Compared with the unmodified sample, the cycling stability of the modified TiO_2_-modified LiNi_0.6_Co_0.2_Mn_0.2_O_2_ samples is significantly improved. When the amount of TiO_2_ coating reaches 5.0%, the 5wt% TiO_2_-NCM material has a discharge capacity of 163.9 mAh g^−1^ after 150 cycles and shows better electrochemical properties with a high cutoff voltage (3.0–4.6 V). The improvement of the cycling performance of TiO_2_-coated NCM is mainly attributed to the following reasons: (1) TiO_2_ has a stable structure, which effectively improves the overall mechanical stability of the material through the close bonding with NCM622; (2) the TiO_2_ coating layer stabilizes the interface between the electrode and the electrolyte and isolates the active substance from the electrolyte. In addition, a small amount of Ti^4+^ is doped into the crystal lattice of NCM622 during the high-temperature roasting process, which maintains the integrity of the crystal structure of NCM622 and plays the role of supporting the structure.

## Figures and Tables

**Table 1:** The lattice parameters (a and c) and I_(003)_/I_(104)_ of the NCM622 and TiO_2_-coated NCM622 samples.

**Sample**	**Lattice Parameters**			**I_(003)_/I_(104)_**
	**a (Å)**	**c (Å)**	**c/a**	
NCM622	2.8619	14.1927	4.9592	1.65
2 wt% TiO_2_-NCM	2.8642	14.2036	4.9590	1.81
5 wt% TiO_2_-NCM	2.8726	14.2497	4.9606	1.83
8 wt% TiO_2_-NCM	2.8769	14.2623	4.9575	1.88

**Table 2:** The discharge specific capacity and coulombic efficiency of NCM622 and TiO_2_-coated NCM samples at 0.3 C.

**Sample**	**Discharge Specific Capacity (mAh g^−1^)**		**Columbic** **Efficiency (%)**
	**1st**	**100th**	
NCM622	179.2	145	80.9
2 wt% TiO_2_-NCM	181.9	151	83.0
5 wt% TiO_2_-NCM	183.5	163.9	89.3
8 wt% TiO_2_-NCM	178.7	135.3	75.7

## Data Availability

The original contributions presented in this study are included in the article/Supplementary Materials. Further inquiries can be directed to the corresponding authors.

## Supplementary Materials

The following supporting information can be downloaded at: https://www.mdpi.com/article/10.3390/ma17246222/s1. Experimental parts: characterizations and electrochemical measurements. Figure S1. Local magnification XRD patterns of peaks (a) (003) and (b) (104). Figure S2. The XPS spectra of bare NCM622: (a) survey spectrum; (b) Ni 2*p*; (c) Co 2*p*; (d) Mn 2*p*. Figure S3. CV curves of NCM622. Figure S4. Coulombic efficiency of NCM622, 2 wt% TiO_2_-NCM, 5 wt% TiO_2_-NCM, and 8 wt% TiO_2_-NCM samples. Table S1. The discharge specific capacity and initial coulombic efficiency of NCM622 and TiO_2_-coated NCM samples at 0.3 C. Table S2. Comparison of electrochemical performances of part of coated LiNi_0.6_Co_0.2_Mn_0.2_O_2_ (NCM) with previous work reported for LIB. Table S3. The Charge transfer resistance (*R_ct_*)of NCM and TiO_2_-coated NCM samples. References [40,41,42] are cited in the Supplementary Materials.

## References

[1] Shi C.M., Takeuchi S., Alexander G.V., Hamann T., O’Neill J., Dura J.A., Wachsman E.D. (2023). High Sulfur Loading and Capacity Retention in Bilayer Garnet Sulfurized-Polyacrylonitrile/Lithium-Metal Batteries with Gel Polymer Electrolytes. Adv. Energy Mater..

[2] Lu J.Y., Xu C., Dose W., Dey S., Wang X.H., Wu Y.H., Li D.P., Ci L.J. (2024). Microstructures of layered Ni-rich cathodes for lthium-ion batteries. Chem. Soc. Rev..

[3] You B., Wang Z., Shen F., Chang Y., Peng W., Li X., Guo H., Hu Q., Deng C., Yang S. (2021). Research progress of sigle-crystal nickel-rich cathode materials for lithium-ion batteries. Small Methods.

[4] Song Y.Z., Wang L., Sheng L., Ren D.S., Liang H.M., Li Y.D., Wang A.P., Zhang H., Xu H., He X.M. (2023). The significance of mitigating crosstalk in lithium-ion batteries: A review. Energy Environ. Sci..

[5] Mei P., Zhang Y., Zhang W. (2023). Low-temperature lithium-ion batteries: Challenges and progressof surface/interface modifications for advanced performance. Nanoscale.

[6] Li J., Zhong W., Deng Q., Zhang Q., Yang C. (2022). Recent progress in synthesis and surface modification of nickel-rich layered oxide cathode materials for lithium-ion batteries. Int. J. Extrem. Manuf..

[7] Moryson Y., Walther F., Sann J., Mogwitz B., Ahmed S., Burkhardt S., Chen L., Klar P.J., Volz K., Fearn S. (2021). Analyzing Nanometer-Thin Cathode Particle Coatings for Lithium-Ion Batteries—The Example of TiO_2_ on NCM622. ACS Appl. Energy Mater..

[8] Wang W.C., Lee C.H., Yu D.N., Kondo Y., Miyahara Y., Abe T., Miyazaki K. (2022). Effects of a Solid Solution Outer Layer of TiO_2_ on the Surface and Electrochemical Properties of LiNi_0.6_Co_0.2_Mn_0.2_O_2_ Cathodes for Lithium-Ion Batteries through the Use ofThin-Film Electrodes. ACS Appl. Energy Mater..

[9] Men L.J., Feng S.Y., Zhang J.F., Luo X.B., Zhou Y.F. (2024). Asyste maticreview of efficientre cycling for the cathode materials of spent lithium-ion batteries: Process inten sification technologies beyond traditional methods. Green Chem..

[10] Jang S.-H., Lee K.-J., Mun J., Han Y.-K., Yim T. (2019). Chemically-induced cathode-electrolyte inter phase created by lithium salt coating on Nickel-rich layered oxides cathode. J. Power Sources.

[11] Marriam I., Tebyetekerwa M., Chathuranga H., Sun K.G., Du A.J., Yan C. (2024). Nickel-Rich Cathode Yarn for Wearable Lithium-Ion Batteries. Adv. Fiber Mater..

[12] Gu H., Mu Y., Zhang S.T., Li Y.Q., Hu H.L., Zhu X.Y., Meng W.J., Qiu J.Y., Ming H. (2024). Enhanced thermal safety and rate capability of nickel-rich cathodes via optimal Nb-doping strategy. Electrochim. Acta.

[13] Li X., Ge W.J., Zhang K.K., Peng G.C., Fu Y.X., Ma X.G. (2022). Comprehensive study of tantalum doping on morphology, structure, and electrochemical performance of Ni-rich cathode materials. Electrochim. Acta.

[14] Zhang X., Belharouak I., Li L., Yu L., Elam J.W., Nie A., Chen X., Yassar R.S., Axelbaum R.L. (2013). Structural and electrochemical study of Al_2_O_3_ and TiO_2_ coated Li_1.2_Ni_0.13_Mn_0.54_Co_0.13_O_2_ cathode material using ALD. Adv. Energ. Mater..

[15] Ge W.J., Fu Y.X., Ma X.G., Li X., Peng G.C. (2022). Dualmodification of LiNi_0.6_Co_0.2_Mn_0.2_O_2_ with MgHPO_4_ as a high-performance cathode material for Li-ion batteries. Energy Adv..

[16] Cao J., Li Y., Wang L.J., Li J., Qiao Y.M., Zhu L.P., Zhang S.N., Yan X.X., Xie H.Q. (2023). Enhanced electrochemical performances of Li_1.2_Ni_0.13_Co_0.13_Mn_0.54_O_2_ cathode material coated with ZrO_2_. Ionics.

[17] Yao L., Liang F., Jin J., Chowdari B.V.R., Yang J.H., Wen Z.Y. (2020). Improved electrochemical property of Ni-rich LiNi_0.6_Co_0.2_Mn_0.2_O_2_ cathode via in-situ ZrO_2_ coating for high energy density lithium-ion batteries. Chem. Eng. J..

[18] Wang L., Liang J., Zhang X., Li S., Wang T., Ma F., Han J., Huang Y., Li Q. (2021). An effectivedual-modification strategytoenhance the performance of LiNi_0.6_Co_0.2_Mn_0.2_O_2_ cathode for Li-ion batteries. Nanoscale.

[19] Yu H., He X.Q., Liang X.H. (2022). AlF_3_-Al_2_O_3_ ALDThin-Film-Coated Li_1.2_Mn_0.54_Co_0.13_Ni_0.13_O_2_ Particles for Lithium-Ion Batteries: Long-Term Protection. ACS Appl. Mater. Interfaces.

[20] Yang H., Zhang H., Zhao W. (2023). Improvement of electrochemical performance of LiNi_0.5_Co_0.2_Mn_0.3_O_2_ byLaF_3_ coating at highcut-offvoltage. Ionics.

[21] Tang W., Peng Z., Shi Y., Sheng X., Shuai H.T., Zhou S., Kong Y., Yan K.P., Lu T.C., Wang G.X. (2019). Enhanced cyclability andsafety performance of LiNi_0.6_Co_0.2_Mn_0.2_O_2_ at elevated temperature by AlPO_4_ modification. J. Alloys Compd..

[22] Zhang W., Liang L., Zhao F., Liu Y., Hou L.R., Yuan C.Z. (2020). Ni-rich LiNi_0.8_Co_0.1_Mn_0.1_O_2_ coated withLi-ion conductive Li_3_PO_4_ as competitive cathodes for high-energy-density lithium-ion batteries. Electrochim. Acta.

[23] Cho W., Kim S.M., Lee K.W., Song J.H., Jo Y.N., Yim T., Kim H., Kim J.-S., Kim Y.-J. (2016). Investigation of new manganese orthophosphate Mn_3_(PO_4_)_2_ coating for nickel-rich LiNi_0.6_Co_0.2_Mn_0.2_O_2_ cathode and improvement of its thermal properties. Electrochim. Acta.

[24] Yang X., Meng Q., Zhang Y.j., Dong P., Fei Z.T., Li C.C., Wang J.J., Li W.B., Li X.F., Xu K.H. (2023). Samariumoxide coating with enhanced lithium storage of regenerated LiNi_0.6_Co_0.2_Mn_0.2_O_2_. Surf. Interfaces.

[25] Venkatachalam P., Duru K.K., Rangarajan M., Sangaraju S., Maram P.S., Kalluri S. (2024). LiNbO_3_ coating on Mg-doped NCM-622 cathode—Adual modification to enhance the electrochemical performance at higher voltage for lithium-ion batteries. J. Solid State Electrochem..

[26] Huang K., Zhou J.X., Yang H.L., Xie T.Z., Lan T., Ong S.C., Jiang H., Zeng Y.B., Guo H., Zhang Y. (2023). TiO_2_–LiF compositecoating for improving NCM622 cathode cycling stability: One-step construction. RSC Adv..

[27] Li W.W., Zhang X.J., Si J.J., Yang J., Sun X.Y. (2021). TiO_2_-coated LiNi_0.9_Co_0.08_Al_0.02_O_2_ cathode materials with enhanced cycle performance for Li-ion batteries. Rare Met..

[28] Wang W.Z., Wu L.Y., Li Z.W., Huang K.S., Jiang J.M., Chen Z.Y., Qi X.D., Dou H., Zhang X.G. (2020). In Situ Tuning Residual Lithium Compounds and Constructing TiO_2_ Coating for Surface Modification of a Nickel-Rich Cathode toward High-Energy Lithium-Ion Batteries. ACS Appl. Energy Mater..

[29] Qin C.C., Cao J.L., Chen J., Dai G.L., Wu T.F., Chen Y.B., Tang Y.F., Li A.D., Chen Y.F. (2016). Improvement of electrochemicalperformance of nickel rich LiNi_0.6_Co_0.2_Mn_0.2_O_2_ cathode active material by ultrathin TiO_2_ coating. Dalton Trans..

[30] You L.Z., Wen Y., Li G.X., Chu B.B., Wu J.H., Huang T., Yu A.S. (2022). Nano-TiO_2_ coated single-crystal LiNi_0.65_Co_0.15_Mn_0.2_O_2_ for lithium-ion batteries with a stable structure and excellent cycling performance at a high cut-off voltage. J. Mater. Chem. A.

[31] Mo Y., Guo L.J., Jin H.F., Du B.D., Cao B.K., Chen Y.G., Li D., Chen Y. (2020). Improved cycling stability of LiNi_0.6_Co_0.2_Mn_0.2_O_2_ through microstructure consolidation by TiO_2_ coating for Li-ion batteries. J. Power Sources.

[32] Chang L.J., Hou Z.L., Yang W., Yang R.F., Wei A.L., Luo S.H. (2025). The theory guides the doping of rare earth elements in the bulk phase of LiNi0.6Co0.2Mn0.2O2 to reach the theoretical limit of energy density. J. Colloid Interface Sci..

[33] Yang W.T., Li R.F., Chen Y., Song L.J., Lu X.Y., Jiang Q. (2023). Preparation of a high performance LiNi_0.6_Co_0.2_Mn_0.2_O_2_ cathode material by using citric acid as a complexing agent. Green Chem..

[34] Jia S.X., Xue J.X., Huo H., Zhou J.J., Li L. (2024). Tailoring the interface of lithium metal batteries with an in situ formed gel polymer electrolyte. J. Mater. Chem. A.

[35] Kim S., Lee K., Kim K., Lee S.S.S., Fortner D., An H., Son Y., Hwang H., Han Y., Myung Y. (2024). Reductive Dissolution of NCM Cathode through Anaerobic Respiration by Shewanella putrefaciens. Environ. Sci. Technol..

[36] Truong B., Wu Y., Hung T. (2021). The effect of lithium-excess on Ni-rich LiNi_0.6_Co_0.2_Mn_0.2_O_2_ cathode materials prepared by a Taylor flow reactor. Electrochim. Acta.

[37] Su M.Y., Dong X.W., Dai X.Y., Huang B.B., Shen M., Xu T., Liu Q.B. (2024). Enhanced Cycling Performance of Spinel LiNi_0.5_Mn_1.5_O_4_ Cathodes through Mg-Mn Hetero-Valent Doping via Microwave Sol-Gel Method. Materials.

[38] Feng Z., Zhang S., Rajagopalan R., Huang X.B., Ren Y.R., Sun D., Wang H.Y., Tang Y.G. (2021). Dual-Element-Modified Single-Crystal LiNi_0.6_Co_0.2_Mn_0.2_O_2_ as a Highly Stable Cathode for Lithium-Ion Batteries. ACS Appl. Mater. Interfaces.

[39] Jo M., Oh P., Kim J., Choi J.H., Kim S., Ha S., Son Y. (2023). Electrochemical lithium storage performance at high voltage and temperature of LiNi_0.6_Co_0.2_Mn_0.2_O_2_ cathode for Lithium-ion batteries by facile Mn_3_(PO_4_)_2_ dry coating. Appl. Surf. Sci..

[40] Huang G.J., Zhong Y., Xia X.H., Wang X.L., Gu C.D., Tu J.P. (2023). Surface-modified and sulfide electrolyte-infiltrated LiNi_0.6_Co_0.2_Mn_0.2_O_2_ cathode for all-solid-state lithium batteries. J. Colloid Interface Sci..

[41] Diao H.H., Jia M.Y., Zhao N., Guo X.X. (2022). LiNi_0.6_Co_0.2_Mn_0.2_O_2_ Cathodes Coated with Dual-Conductive Polymers for High-Rate and Long-Life Solid-State Lithium Batteries. ACS Appl. Mater. Interfaces.

[42] Liu W.M., Zeng S.S., Wang P.P., Huang J., Shen B., Qin M.L., Wang W.G., Tang Z.X. (2024). Dual-coated single-crystal LiNi_0.6_Co_0.2_Mn_0.2_O_2_ as high-performance cathode materials for lithium-ion batteries. J. Solid State Electrochem..

