# Supplementary material for: Effect of TiO2 Coating on Structure and Electrochemical Performance of LiNi0.6Co0.2Mn0.2O2 Cathode Material for Lithium-Ion Batteries
Source: Materials (Basel). 2024 Dec 19;17(24):6222. doi: 10.3390/ma17246222 (PMC11679795; doi:10.3390/ma17246222)
Supplement: Supplementary file 1 [file materials-17-06222-s001.zip › materials-3315461-supplementary.pdf]

Electronic Supplementary Information

Effect of TiO_2 coating on structure and electrochemical performance of $LiNi_{0.6}Co_{0.2}Mn_{0.2}O_2$ cathode material for lithium-ion batteries

Lin Li[1,#], Zhongyu Li[2,#], Zhifan Kuang[2], Hao Zheng[b], Minjian Yang[1], Jianwen Liu[2], Shiquan Wang[2,3*], Hongying Liu[2,3*]

[1] School of Chemical Engineering, Guizhou University of Engineering Science, Bijie 551700, P. R. China; lilin_bj2024@163.com (L. L.), honglinymj@163.com (M. Y.)

[2] Collaborative Innovation Center for Advanced Organic Chemical Materials Co-constructed by the Province and Ministry & Ministry of Educational Key Laboratory for the Synthesis and Application of Organic Functional Molecules & College of Chemistry and Chemical Engineering, Hubei University, Wuhan, 430062, P. R. China; 936414585@qq.com (Z. L.); 2205454832@qq.com (Z. K.); zhengaho1986@126.com (H. Z.); jianwen@hubu.edu.cn (J. L.); wsqhao@hubu.edu.cn (S. W.); liuhy@hubu.edu.cn (H. L.)

[3] Hubei Three Gorges Laboratory, Yichang 443008, P.R. China

# Lin Li and Zhongyu Li contributed equally to this work.

* Correspondence: lilin_bj2024@163.com (L. L.), wsqhao@hubu.edu.cn (S. W.), liuhy@hubu.edu.cn (H. L.)

Experimental

Characterizations

The crystal structures of NCM622 and TiO_2-coated NCM600 samples were characterized by X-ray powder diffraction (XRD) (Bruker AXS, D8 diffractometer, Germany) using a Cu-Kα source from 10 to 70°. The morphology and microstructure of the samples were observed by transmission electron microscopy (TEM) (Thermo Scientific Talos F200S G2 200kV), scanning electron microscopy (SEM) (JEOL JSM-7100F, Japan), and X-ray (EDX) detector (Oxford Instruments, INCA). The chemical states of Ni, Co, Mn, O, Ti, and C were characterized by X-ray photoelectron spectroscopy (XPS) (VGESCA-LABMK II).

Electrochemical measurements

The electrochemical performance of the samples (NCM622 and TiO_2-coated NCM622 samples) was evaluated using CR2025 coin-type half-cells. The electrodes were prepared by mixing the NCM622 and TiO_2-coated NCM samples (80 wt. %), carbon black (BP2000) (10 wt.%), and polyvinyldifluoride (PVDF) (10 wt. %) with a moderate amount of NMP and pasting onto Al foil. The electrolyte was 1 mol L^{-1} $LiPF_6$ in a mixture of diethyl carbonate (DEC), ethylidene carbonate (EC), and dimethyl carbonate (DMC) (1:1:1 by volume). The coin-type cells were assembled in an Ar-filled glove box using metal Li foil as a counter electrode and diaphragm as the separator. For the charge and discharge test, an automatic battery test system (Neware, China) with different voltage ranges of 3.0~4.3 V and 3.0~4.6 V was used. Electrochemical impedance spectroscopy (EIS, the range of 0.01 Hz-100 KHz, the amplitude of 5 mV) and cyclic voltammetry (CV, the voltage range of 3.0~4.3 V, the scan rate of 0.1 mV/s) measurements of the cells were performed on an electrochemical workstation (CHI 660E, China).

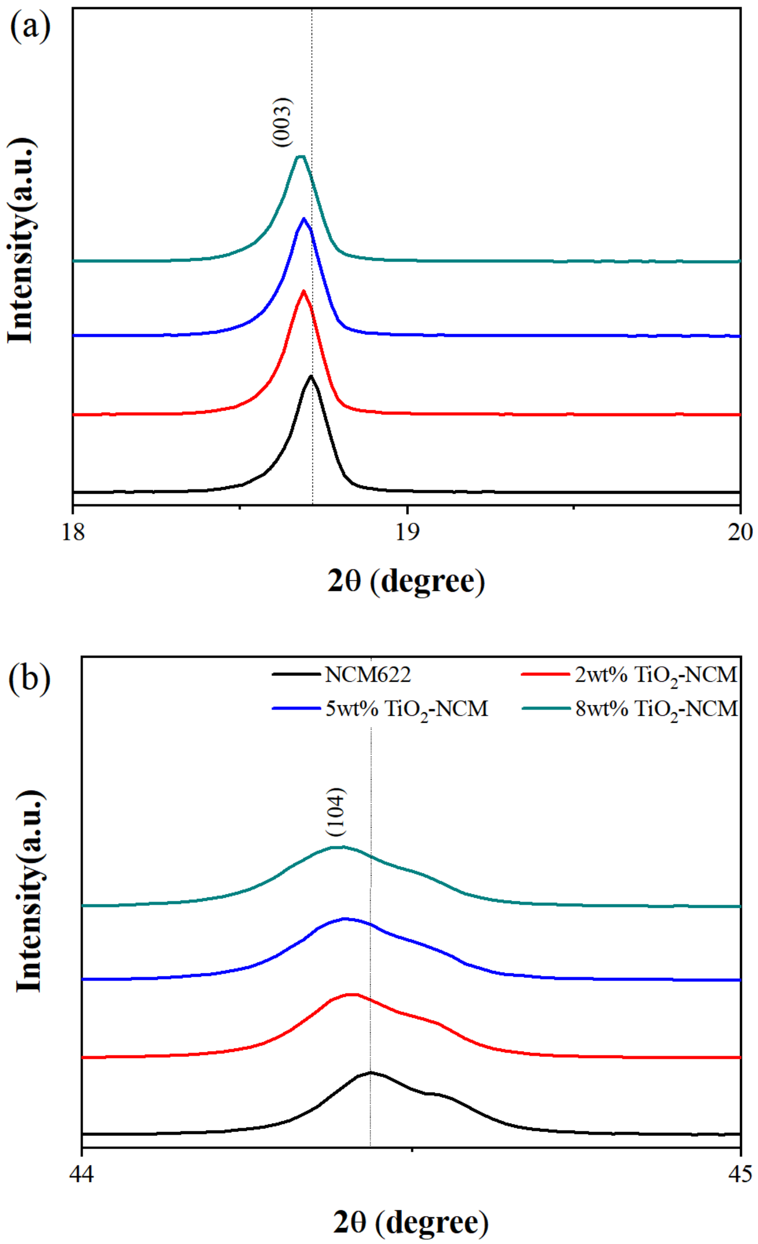

Figure S1. Local magnification XRD patterns of peaks (a) (003) and (b) (104).

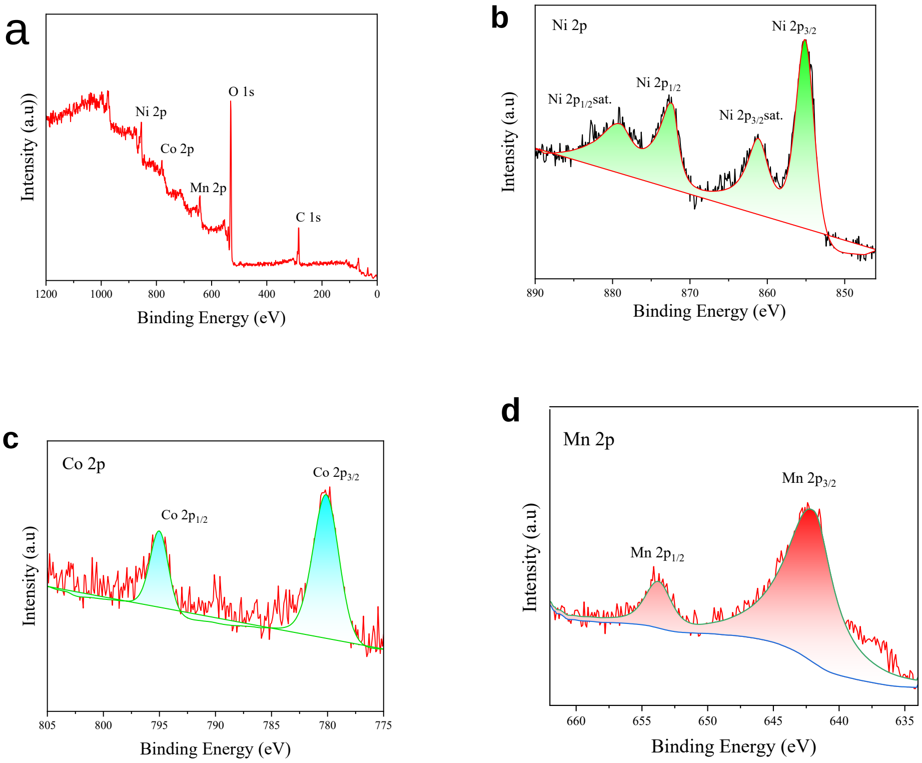

Figure S2. The XPS spectra of bare NCM622: (a) survey spectrum; (b) Ni 2*p*; (c) Co 2*p*; (d) Mn 2*p*.

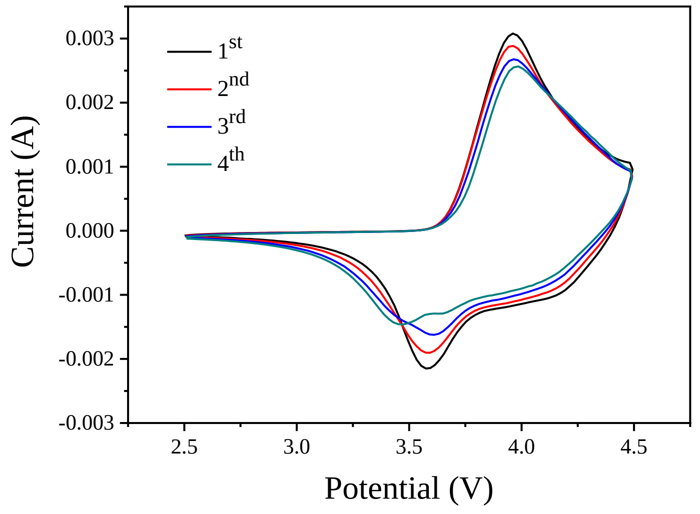

Figure S3. CV curves of NCM622.

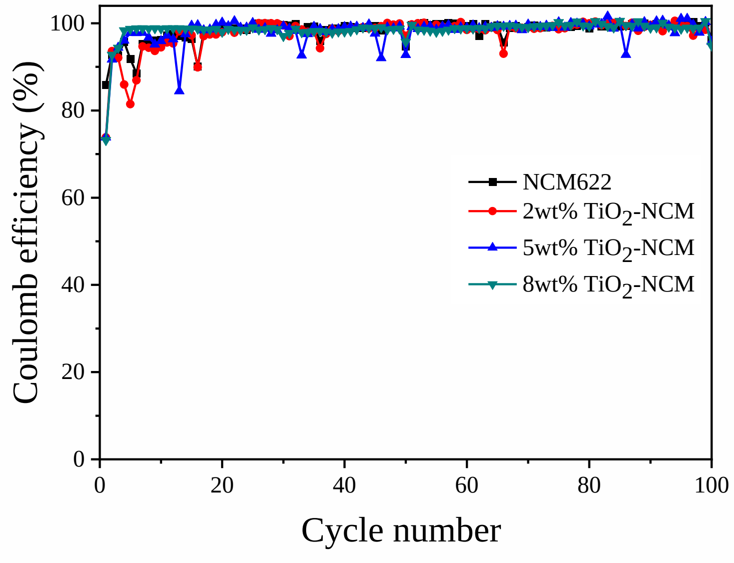

Figure S4. Coulombic efficiency (CE) of NCM622, 2 wt% TiO_2-NCM, 5 wt% TiO_2-NCM, and 8 wt% TiO_2-NCM samples.

Table S1. The discharge-specific capacity and initial coulombic efficiency of NCM622 and TiO_2-coated NCM samples at 0.3 C.

Sample	Discharge Specific Capacity (mAh g^{-1})		Initial Columbic Efficiency (%)
	1st	100th	
NCM622	179.2	145	80.9
2 wt% TiO_2-NCM	181.9	151	83.0
5 wt% TiO_2-NCM	183.5	163.9	89.3
8 wt% TiO_2-NCM	178.7	135.3	75.7

Table S2 Comparison of electrochemical performances of part of TiO_2-coated $LiNi_{0.6}Co_{0.2}Mn_{0.2}O_2$ (NCM) with previous work reported for LIBs.

Coating material	Cutoff voltage (V) / Rate (C)	Cycles	Capacity (mAh g^{-1}) / Retention (%)
Samarium oxide [1]	2.8–4.3 V / 0.1 C		167.56
$Mn_3(PO_4)_2$ [2]	4.55 V		
$Li_{1.5}Al_{0.5}Ge_{1.5}(PO_4)_3$ [3]	0.05 C	100	141.5
$MgHPO_4$ [4]	0.1C / 3-4.3	200	88.3
PEG/PANI [5]	0.1 C / 3–4.3 V	100	158 / 88
ZnO and $AlPO_4$ [6]	1 C / 2.75 – 4.5 V	100	91.2
$Li_4Ti_5O_{12}$ [7]	0.1 C		183.0
$LiNbO_3$ coating and Mg [8]	4.3 V		85.4
TiO_2–LiF [9]	1C / 2.7–4.3 V	200	79.7
5wt% TiO_2-NCM (This work)	0.3 C/ 3-4.3	100	163.8 / 89.3
	0.5 C/3-4.6	150	107.3/53.5

Table S3 The charge transfer resistance (R_{ct}) of NCM and TiO_2-coated NCM samples.

Sample	NCM622	2 wt% TiO_2-NCM	5 wt% TiO_2-NCM	8 wt% TiO_2-NCM
EIS(Ω)	191	142	113	258

1. Yang, X.; Meng, Q.; Zhang, Y.j.; Dong, P.; Fei, Z.T.; Li, C.C.; Wang, J.J.; Li, W.B.; Li, X.F.; Xu, K.H.; Zhang, K. Samarium oxide coating with enhanced lithium storage of regenerated $LiNi_{0.6}Co_{0.2}Mn_{0.2}O_2$. Surf. Interfaces. 2023, 42, 103405.

2. Jo, M.; Oh, P.; Kim, J.; Choi, J.H.; Kim, S.; Ha, S.; Son, Y. Electrochemical lithium storage performance at high voltage and temperature of $LiNi_{0.6}Co_{0.2}Mn_{0.2}O_2$ cathode for Lithium-ion batteries by facile Mn3(PO4)2 dry coating. Appl. Surf. Sci. 2023, 613, 156018

3. Huang, G.J.; Zhong, Y.; Xia, X.H.; Wang, X.L.; Gu, C.D.; Tu, J.P. Surface-modified and sulfide electrolyte-infiltrated $LiNi_{0.6}Co_{0.2}Mn_{0.2}O_2$ cathode for all-solid-state lithium batteries. J. Colloid Interface Sci. 632, 2023, 11-18

4. Ge, W.J.; Fu, Y.X.; Ma, X.G.; Li, X.; Peng, G.C. Dual modification of $LiNi_{0.6}Co_{0.2}Mn_{0.2}O_2$ with MgHPO4 as a high-performance cathode material for Li-ion batteries. Energy Advances 1, 2022, 28-37

5. Diao, H.H.; Jia, M.Y.; Zhao, N.; Guo, X.X. $LiNi_{0.6}Co_{0.2}Mn_{0.2}O_2$ Cathodes Coated with Dual-Conductive Polymers for High-Rate and Long-Life Solid-State Lithium Batteries. ACS Appl. Mater. Interfaces 2022, 14, 21, 24929 − 24937

6. Wang, W.C.; Lee, C.; Yu, D.N.; Kondo, Y.; Miyahara, Y.; Abe, T.; Miyazaki, K. Effects of a Solid Solution Outer Layer of TiO2 on the Surface and Electrochemical Properties of $LiNi_{0.6}Co_{0.2}Mn_{0.2}O_2$ Cathodes for Lithium-Ion Batteries through the Use of Thin-Film Electrodes. ACS Appl. Energy Mater. 2022, 5, 5117-5126

7. Liu, W.M.; Zeng, S.S.; Wang, P.P.; Huang, J.; Shen , B.; Qin , M.L.; Wang, W.G.; Tang, Z.X. Dual-coated single-crystal $LiNi_{0.6}Co_{0.2}Mn_{0.2}O_2$ as high-performance cathode materials for lithium-ion batteries. J. Solid State Electrochem (2024). https://doi.org/10.1007/s10008-024-06048-5

8. Venkatachalam, P.; Duru, K.K.; Rangarajan, M.; Sangaraju, S.; Maram, P.S.; Kalluri, S. $LiNbO_3$ coating on Mg-doped NCM-622 cathode—a dual modification to enhance the electrochemical performance at higher voltage for lithium-ion batteries. J. Solid State Electrochem. 2024, 28, 3509–3515,

9. Huang, K.; Zhou, J.X.; Yang, H.L.; Xie, T.Z.; Lan, T.; Ong, S.C.; Jiang, H.; Zeng, Y.B.; Guo, H.; Zhang, Y. TiO_2–LiF composite coating for improving NCM622 cathode cycling stability: one-step construction. RSC Adv. 2023, 13, 33905–33910.
